# Association between Duration of Carbon Dioxide Pneumoperitoneum during Laparoscopic Abdominal Surgery and Hepatic Injury: A Meta-Analysis

**DOI:** 10.1371/journal.pone.0104067

**Published:** 2014-08-11

**Authors:** Hao Lai, Xianwei Mo, Yang Yang, Jun Xiao, Ke He, Jiansi Chen, Yuan Lin

**Affiliations:** 1 Department of Gastrointestinal Surgery, Affiliated Cancer Hospital of Guangxi Medical University, Nanning, Guangxi Autonomous Region, China; 2 Department of Neck and Head Surgery, Affiliated Cancer Hospital of Guangxi Medical University, Nanning, Guangxi Autonomous Region, China; University Hospital Heidelberg, Germany

## Abstract

**Background:**

The aim of this study is to accurately assess whether the duration of intraoperative carbon dioxide pneumoperitoneum (CDP) is associated with the induction of hepatic injury.

**Methods:**

We conducted a systematic review of PubMed, Embase, and Cochrane Library databases (through February 2014) to identify case-match studies that compared high-pressure CDP with low-pressure CDP or varied the duration of CDP in patients who underwent abdominal surgery. The outcome of interest was postoperative liver function (ALT, AST, TB).

**Results:**

Eleven comparative studies involving 2,235 participants were included. Overall, levels of ALT, AST, and TB (on postoperative days 1, 3, and 7) were significantly elevated in the study groups. However, the results of the subanalyses of those who underwent laparoscopic colorectal cancer resection (LCR) versus open colorectal cancer resection (OCR) and those who underwent laparoscopic gastric bypass (LGBP) versus open gastric bypass (OGBP) were inconsistent.

**Conclusions:**

The current evidence suggests that the duration of CDP during laparoscopic abdominal surgery may be associated with hepatic injury. Additional large-scale, randomized, controlled trials are urgently needed to further confirm this.

## Introduction

Laparoscopic surgery is one of the most significant surgical advances of the twentieth century. Its numerous advantages are well known: shorter hospital stays and convalescence, limited postoperative pain, more rapid recovery, and a reduction in complications and lost working days [1], [2], [3], [4]. However, these clinical advantages must be compared with the effects caused by the pressure and duration of carbon dioxide pneumoperitoneum (CDP) on liver function during laparoscopic abdominal surgery.

With the increasing use of laparoscopic surgery, new concerns have arisen regarding the effects of CDP on cardiovascular and respiratory systems. An important hemodynamic change is the transient reduction in hepatic blood flow caused by CDP. In 1994, Halevy et al. [5] were the first to report that the pressure created by CDP and its duration influence the degree of hepatic ischemia and can cause liver enzyme elevation.

To date, conflicting results have been reported regarding the effects of CDP on splanchnic and liver perfusion. Most reports have suggested that there is a direct correlation between duration of CDP and hepatic injury [6], [7], [8], [9], [10], [11], [12], but several other retrospective analyses have reported *no* effect [13], [14], [15], [16]. These differences leave uncertainty in the surgical field regarding the effects of CDP on the induction of hepatic injury. Thus, it is important to assess all the available data and review controversial or inconclusive results.

We assumed that the duration of CDP would be associated with hepatic injury in those who underwent laparoscopic abdominal surgery. Then, we performed a systematic meta-analysis to evaluate this assumption.

## Materials and Methods

### Search strategy

The Pubmed, Embase, and Cochrane Library electronic databases were searched for comparative studies published up to February 2014. The following medical subject heading (MeSH) terms and words were used for the search in all possible combinations: “pneumoperitoneum,” “insufflation,” “aeroperitoneum,” “liver function,” and “hepatic function.” A filter for identifying comparable studies recommended by the Cochrane Collaboration was used to filter out non-randomized studies in Pubmed and Embase [17]. A manual search of the reference lists of relevant articles was also performed. No language or time restriction was used. Data were extracted from each study by two independent reviewers (Hao Lai and Xianwei Mo). Disagreements were resolved by consensus.

### Eligibility criteria

Studies were considered eligible if they met the following inclusion criteria: Study design—case-match design (random controlled trials or controlled clinical trials); population—patients undergoing laparoscopic abdominal surgery; intervention—some period of CDP in the intraoperative period; comparator—patients undergoing laparoscopic abdominal surgery with lower pressure CDP or patients undergoing open abdominal surgery; and all included studies had to report at least one of the following postoperative outcome measures: ALT, AST, TB, ALP, GGT, and Alb.

Studies were excluded if any of the following criteria were met: not a case-match design, animal experiment, primary outcome was not the one of interest, interventions other than duration of CDP, raw data could not be extracted in an appropriate format and could not be obtained from the authors or other published results.

### Data extraction

Data were independently extracted from all eligible publications by two investigators (Hao Lai and Xianwei Mo) according to the aforementioned inclusion criteria. Disagreements were resolved by discussion during a consensus meeting with a third reviewer (Yuan Lin). Data extracted included first author's last name, year of publication, study design, country, type of surgery, patient population, type of CDP, and postoperative results of liver function (ALT, AST, TB, ALP, GGT, LDH, and Alb).

Outcome variables were considered suitable for analysis if they met the following criteria: continuous outcomes were reported as means and standard deviations, and the same variables were reported for a minimum of five comparison groups and a minimum of two days postoperatively. Three outcome variables were considered the most suitable for analysis: postoperative results for ALT (days 1, 3, 7), AST (days 1, 3, 7), and TB (days 1, 3, 7).

### Risk of bias assessment

Risk of bias was evaluated by the reviewers using the Cochrane Handbook for Systematic Reviews of Interventions [17]. The assessment was based on sequence generation, allocation concealment, blinding, incomplete outcome data, selective outcome reporting, and other sources of bias. Agreement was achieved through discussion when necessary.

### Statistical analysis

Statistical analysis for continuous variables was performed using the standard mean difference (SMD) and a random-effects or fixed-effects model was used depending on the presence or absence of heterogeneity. We used the *Q*-based chi-square test and the *I^2^* statistic to assess heterogeneity between studies, with a *P*-value of less than 0.10 representing statistical significance. Sensitivity and subgroup analyses were used to explore potential causes of heterogeneity. Subgroup analyses were performed to examine whether hepatic injury varied by duration of CDP and type of surgery. Publication bias was also evaluated by constructing a funnel plot with a visual assessment of asymmetry [18], [19]. All analyses were performed using STATA software (ver. 9.0; Stata Corp, College Station, TX).

## Results

### Literature search

**Figure 1:**
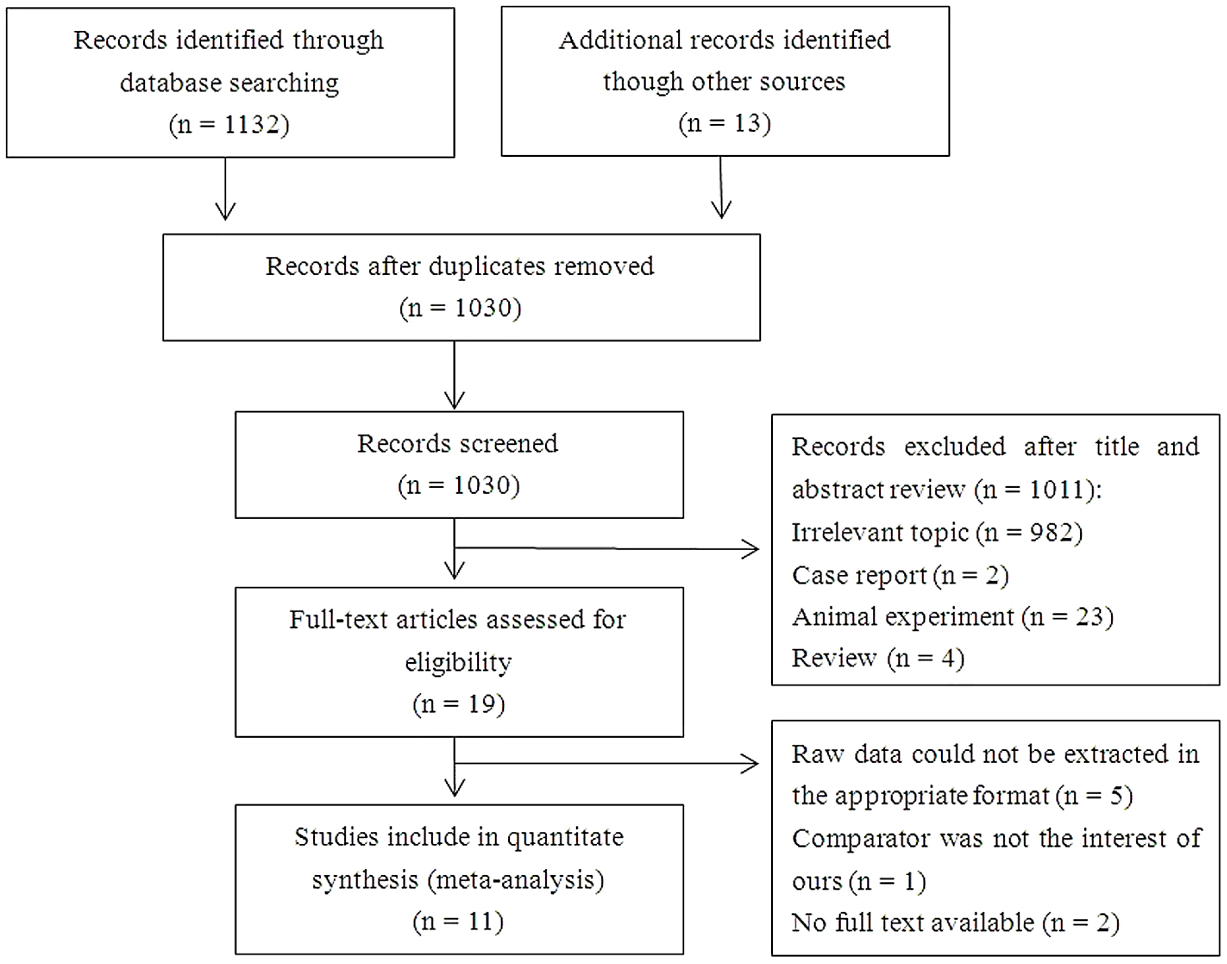
Flow chart for the systematic search and study selection strategy.

Figure 1 depicts a PRISMA flow chart for study inclusion and exclusion. A total of 1,132 records were retrieved from the database search and 13 were identified through manual searches of the reference lists of relevant articles. After omitting duplicate results, 1,030 records remained. Of these, 19 were selected for full-text examination. Eight of these were then excluded for the following reasons: raw data could not be extracted in an appropriate format (*n* = 5) [20], [21], [22], [23], [24], the comparator was not of interest (*n* = 1) [12], or no full text was available (*n* = 2) [25], [26]. Eleven studies fulfilled the inclusion criteria and were included in the meta-analysis [6], [7], [8], [9], [10], [11], [13], [14], [15], [16], [27].

### Study characteristics

The characteristics of the 11 studies included, which were published between 2003 and 2013, are presented in Table 1. Six studies used randomized controlled trials [6], [7], [9], [10], [14], [27], and the other five used controlled clinical trials [8], [11], [13], [15], [16]. One study was conducted in China [6], one in India [7], three in Turkey [8], [10], [27], one in Bosnia and Herzegovina [9], two in Korea [13], [16], two in Japan [11], [15], and one in the United States [14]. There was a total of 1,539 patients were in the study group and 696 in the control group. The operations performed included cholecystectomy, colorectal cancer resection, distal gastrectomy, and gastric bypass. In three studies [7], [9], [10], laparoscopic techniques were performed with standard-pressure CDP (12–14 mmHg) in the study group and low-pressure CDP (7–10 mmHg) in the control group. In eight studies [6], [8], [11], [13], [14], [15], [16], [27], the CDP pressure ranged from 8 to 15 mmHg in the study group, whereas it was zero (i.e., open surgery) in the control group. Among the eight studies that described the inclusion criteria for patients [6], [7], [8], [9], [10], [11], [13], [14], seven of them presented preoperative normal serum liver enzyme values as the most common reason for inclusion [6], [7], [8], [9], [10], [11], [14]; the remaining studies included patients with any disease that might cause liver enzymes to be elevated preoperatively [13]. In the eight studies describing the exclusion criteria for patients, the most common reason for exclusion was the presence of any condition that might cause serum liver enzymes to be elevated in the preoperative period [6], [7], [8], [10], [14], [15], [16], [21], [27]. The outcome measures of these studies varied and included some index of postoperative liver function from days 1 to 7.

**Table 1:** Characteristics of included studies.

First author	Study design	Country	Patients population	Type of surgery	Type of CDP	Inclusion criteria	Exclusion criteria
Tan2003	RCT	China	143 VS 40 143 VS 40 18 VS 23	LC VS OC (group 1) LC VS OC (group 2) LCR VS OCR	12–14 mmHg VS open 12–14 mmHg VS open 12–14 mmHg VS open	Patients had normal values of serum liver enzymes preoperative	Patients who underwent ERCP or EST within one week preoperative; Patients who developed complications which might cause serum liver enzymes elevated
Gupta2013	RCT	India	51 VS 50	LC VS LC	14 mmHg VS 8 mmHg	Patients with normal preoperative LFTs	Patients with a history of jaundice, with common bile duct stones, or with abnormal preoperative LFTs
Güven2007	CCT	Turkey	267 VS 54	LC VS OC	12–14 mmHg VS open	Patients with fully completed data on results of liver function test	Patients who with disease which might cause serum liver enzymes elevated preoperative
Hasukic2005	RCT	Tuzla	25 VS 25	LC VS LC	14 mmHg VS 7 mmHg	Patients without history of previous liver diseases and exhibited normal values on preoperative liver function tests.	Patients who with disease which might cause serum liver enzymes elevated preoperative; age ≤18 years, pregnancy and lactation, previous extensive abdominal surgery, and an ASA grade of 3 or more.
Eryılmaz2012	RCT	Turkey	23 VS 20	LC VS LC	14 mmHg VS 10 mmHg	ASA physical status I or II patients	Patients with liver failure, coagulopathy, and known allergy to medication drugs
Sakorafas2005	RCT	Turkey	72 VS 36	LC VS OC	14 mmHg VS open	NA	Patients who with disease or complication which might cause serum liver enzymes elevated in perioperative period
Jeong2011	CCT	Korea	91 VS 124 43 VS 78	LADG VS ODG LCR VS OCR	12 mmHg VS open 12 mmHg VS open	NA	Patients underwent any surgical technique which might affect their liver function during surgery.
Kinjo2011	CCT	Japan	199 VS/120 324 VS 56	LADG VS ODG LCR VS OCR	8 mmHg VS open 8 mmHg VS open	NA	Patients with liver tumor, hepatic disease or whose liver function were abnormal preoperative
Yoon2011	CCT	Korea	18 VS 32	LADG VS ODG	12 mmHg VS open	Liver cirrhosis patients; fatty liver patients, healthy hepatitis B virus carriers; and healthy hepatitis C virus carriers.	NA
Etoh2007	CCT	Japan	147 VS 58	LADG VS ODG	10 mmHg VS open	LADG was selected for tumors limited to the mucosal layer or superficial submucosal layer and ODG for those invading deep submucosal layer.	NA
Nguyen2003	RCT	USA	18 VS 18	LGBP VS OGBP	15 mmHg VS open	Patients being evaluated for surgical treatment of morbid obesity	Patients who had undergone previous obesity or gastric surgery, had a large abdominal ventral hernia, or a history of cirrhosis

Note: ASA: American Society of Anesthesiology; LC: laparoscopic cholecystectomy; OC: open cholecystectomy; LCR: laparoscopic colorectal cancer resection; OCR: open colorectal cancer resection; LADG: laparoscopic assisted distal gastrectomy; OADG: open assisted distal gastrectomy; LGBP: laparoscopic gastric bypass; OGBP: open gastric bypass; NA: not available;

### Quantitative synthesis of data

#### ALT

**Figure 2:**
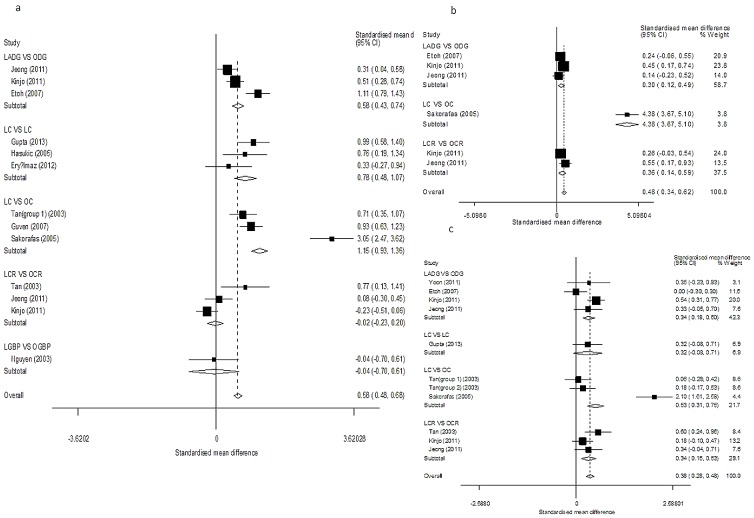
Forest plots of postoperative ALT results in subgroup analysis. (a: day 1; b: day 3; c: day 7).

(Fig. 2. Table 2A) Ten studies described ALT results on postoperative day 1 [6], [7], [8], [9], [10], [11], [14], [15], [16], [27]. ALT was not significantly elevated in the laparoscopic colorectal cancer resection (LCR) versus open colorectal cancer resection (OCR) (*SMD* = −0.02, 95% CI = −0.23–0.20, *P* = 0.86; *I^2^* = 75.7% and *P_Q_* = 0.02 for heterogeneity) or the laparoscopic gastric bypass (LGBP) versus open gastric bypass (OGBP) (*SMD* = −0.04, 95% CI = −0.70–0.61, *P* = 0.90) subanalyses, but other subgroups showed inconsistent results. The overall pooled estimate results showed significant ALT elevations in the study groups (*SMD* = 0.58, 95% CI = 0.48–0.68, *P*<0.01; *I^2^* = 91.3% and *P_Q_*<0.01 for heterogeneity).

**Table 2:** Results of pools SMDs and test for heterogeneity.

a		Test for heterogeneity			b		Test for heterogeneity			c		Test for heterogeneity		
Subgroups	Pools SMDs	*P*	*I^2^*	*P_Q_*	Subgroups	Pools SMDs	*P*	*I^2^*	*P_Q_*	Subgroups	Pools SMDs	*P*	*I^2^*	*P_Q_*
Results for postoperative day 1					Results for postoperative day 1					Results for postoperative day 1				
LADG vs. ODG	0.58 (0.43, 0.74)	0.00	86.2%	0.00	LADG vs. ODG	0.51 (0.36, 0.66)	0.00	84.4%	0.00	LADG vs. ODG	0.34 (0.18, 0.51)	0.00	0%	0.50
LC vs. LC	0.78 (0.48, 1.07)	0.00	34.9%	0.22	LC vs. LC	0.82 (0.52, 1.11)	0.00	69.7%	0.04	LC vs. LC	0.28(−0.01, 0.56)	0.06	34.9%	0.22
LC vs. OC	1.15 (0.93, 1.36)	0.00	96%	0.00	LC vs. OC	0.97 (0.76, 1.19)	0.00	97.4%	0.00	LCR vs. OCR	0.11(−0.12, 0.33)	0.35	34.2%	0.22
LCR vs. OCR	−0.02 (−0.23, 0.20)	0.86	75.7%	0.02	LCR vs. OCR	0.05 (−0.16, 0.26)	0.64	0%	0.43	Overall	0.26 (0.15, 0.39)	0.00	12.6%	0.33
LGBP vs. OGBP	−0.04(−0.70, 0.61)	0.90	-	-	LGBP vs. OGBP	−0.21(−0.87,0.61)	0.44	-	-					
Overall	0.58 (0.48, 0.68)	0.00	91.3%	0.00	Overall	0.53 (0.43, 0.63)	0.00	91.6%	0.00					
Results for postoperative day 3					Results for postoperative day 3					Results for postoperative day 3				
LADG vs. ODG	0.31 (0.12, 0.49)	0.00	0%	0.39	LADG vs. ODG	0.35 (0.19, 0.50)	0.00	85.4%	0.00	LADG vs. ODG	0.38(0.22, 0.55)	0.00	0%	0.55
LC vs. OC	4.39 (3.67, 5.10)	0.00	-	-	LC vs. OC	4.43 (3.71, 5.14)	0.00	-	-	LCR vs. OCR	0.08(−0.15, 0.30)	0.50	0%	0.37
LCR vs. OCR	−0.36 (−0.14, 0.59)	0.00	33.1%	0.22	LCR vs. OCR	0.09 (−0.14, 0.32)	0.44	0%	0.83	Overall	0.39 (0.27, 0.52)	0.00	38.6%	0.16
Overall	0.48 (0.34, 0.62)	0.00	95.9%	0.00	Overall	0.39 (0.27, 0.52)	0.00	96.5%	0.00					
Results for postoperative day 7					Results for postoperative day 7					Results for postoperative day 7				
LADG vs. ODG	0.34 (0.18, 0.50)	0.00	61.2%	0.05	LADG vs. ODG	0.09(−0.06, 0.23)	0.00	0%	0.87	LADG vs. ODG	0.22(0.06, 0.38)	0.01	73%	0.01
LC vs. LC	0.32 (−0.08, 0.71)	0.11	-	-	LC vs. LC	0.35 (−0.04, 0.74)	0.08	-	-	LC vs. LC	−0.06(−0.45,0.34)	0.78	-	-
LC vs. OC	0.53 (0.31, 0.75)	0.00	96%	0.00	LC vs. OC	0.41 (0.19, 0.63)	0.00	96.7%	0.00	LCR vs. OCR	0.18(−0.05, 0.41)	0.12	76.8%	0.04
LCR vs. OCR	0.34 (0.15, 0.53)	0.00	38.3%	0.20	LCR vs. OCR	0.12 (−0.10, 0.33)	0.29	31.6%	0.23	Overall	0.18 (0.06, 0.30)	0.00	64.8%	0.01
Overall	0.38 (0.28, 0.48)	0.00	84.1%	0.00	Overall	0.53 (0.43, 0.63)	0.00	85.9%	0.00					

Four studies evaluated ALT results on postoperative day 3 [11], [15], [16], [27] and seven studies evaluated them on postoperative day 7 [6], [11], [13], [14], [15], [16], [27]. Subgroup analyses showed significant elevations of ALT in all subgroups, and the overall estimated results supported this finding.

**Figure 3:**
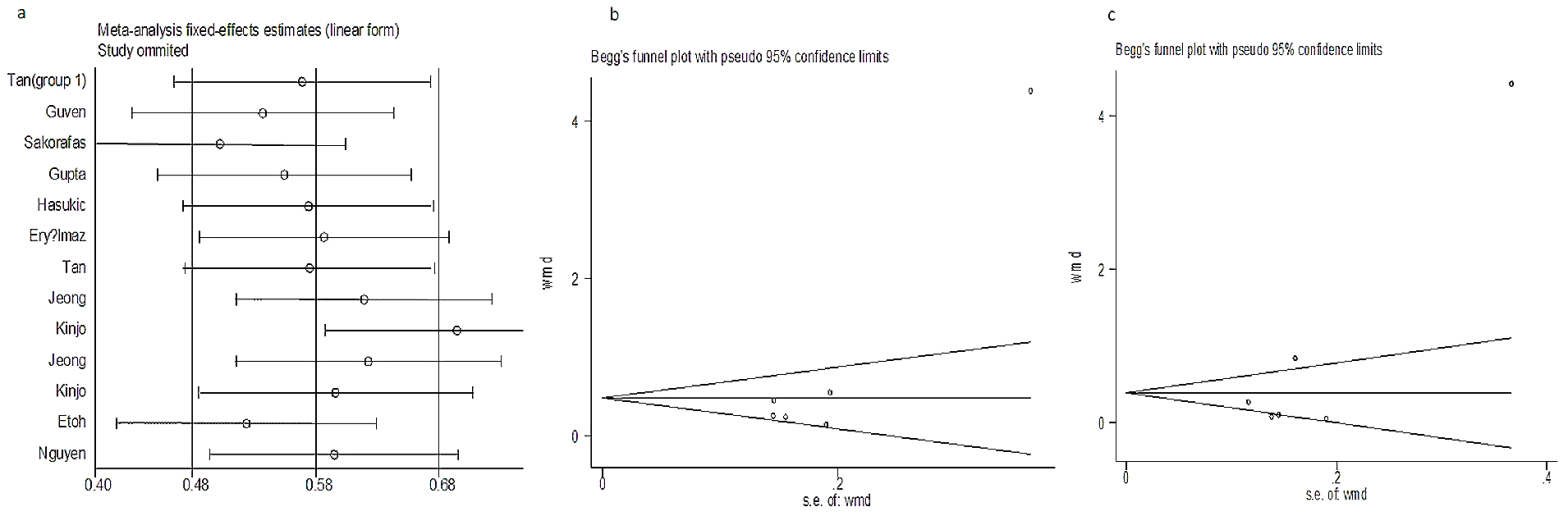
a. Sensitivity analysis. b. Funnel plot analysis to detect publication bias for ALT results on postoperative day 3; each point represents a separate study for the indicated association. c, Funnel plot analysis to detect publication bias for AST results on postoperative day 3; each point represents a separate study for the indicated association.

In the analyses above, there was a high degree of heterogeneity across trials. Publication bias was not evident in the results of postoperative day 1 (*P* = 0.30) or day 7 (*P* = 0.36), but it was observed in the results of postoperative day 3 (*P* = 0.02, Fig. 3B).

#### AST

**Figure 4:**
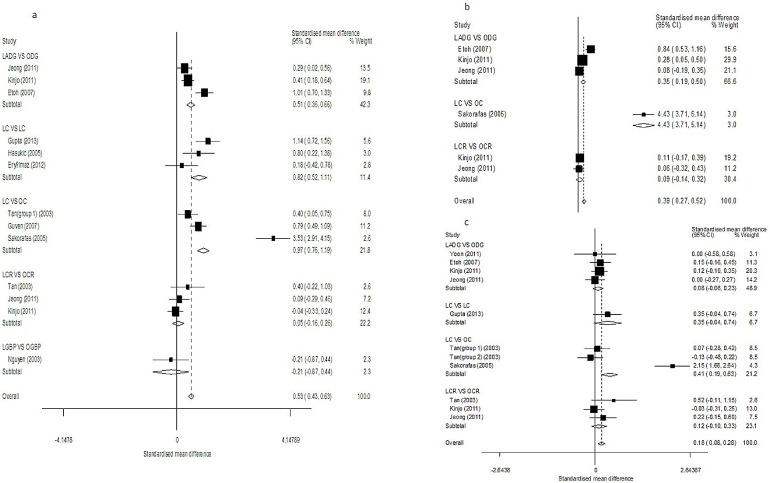
Forest plots of postoperative AST results in subgroup analysis. (a: day 1; b: day 3; c: day 7).

(Fig. 4, Table 2B) Ten studies evaluated AST results on postoperative day 1 [6], [7], [8], [9], [10], [11], [14], [15], [16], [27]. Subgroup analyses showed significant differences in all subgroup comparisons, except for LGBP versus OGBP (*SMD* = −0.21, 95% CI = −0.87–0.61, *P* = 0.44) and LCR versus OCR (*SMD* = 0.05, 95% CI = −0.16–0.26, *P* = 0.64; *I^2^* = 0% and *P_Q_* = 0.43 for heterogeneity). The overall pooled estimates showed significant differences between the study and control groups (*SMD* = 0.53, 95% CI = 0.43–0.63, *P*<0.01; *I^2^* = 91.6% and *P_Q_*<0.01 for heterogeneity).

Results on postoperative day 3 were reported in four studies [11], [15], [16], [27]. Subgroup analysis continued to show significant elevations in AST in all treatment groups analyses the except LCR versus OCR comparison (*SMD* = 0.09, 95% CI = −0.14–0.32, *P* = 0.44; *I^2^* = 0% and *P_Q_* = 0.83 for heterogeneity), and the overall estimates revealed significant differences between the study and control groups (*SMD* = 0.39, 95% CI = 0.27–0.52, *P*<0.01; *I^2^* = 96.5% and *P_Q_*<0.01 for heterogeneity).

Eleven studies evaluated AST results on postoperative day 7 [6], [7], [8], [9], [10], [11], [13], [14], [15], [16], [27]. Most studies showed that AST was significantly elevated in the high-pressure CDP groups, and the overall estimates also supported this (*SMD* = 0.53, 95% CI = 0.43–0.63, *P*<0.01; *I^2^* = 85.9% and *P_Q_*<0.01 for heterogeneity). However, no significant differences were found in the LC versus OC (*SMD* = 0.35, 95% CI = −0.04 subgroup 0.74, *P* = 0.08) and LCR versus OCR (*SMD* = 0.12, 95% CI = −0.10 subgroup 0.33, *P* = 0.29; *I^2^* = 31.6% and *P_Q_* = 0.23 for heterogeneity) subgroup analyses.

In these analyses, there was evidence of significant heterogeneity across the trials. No publication bias was detected, with the exception of the results for postoperative day 3 (P = 0.04, Fig. 3C).

#### TB

**Figure 5:**
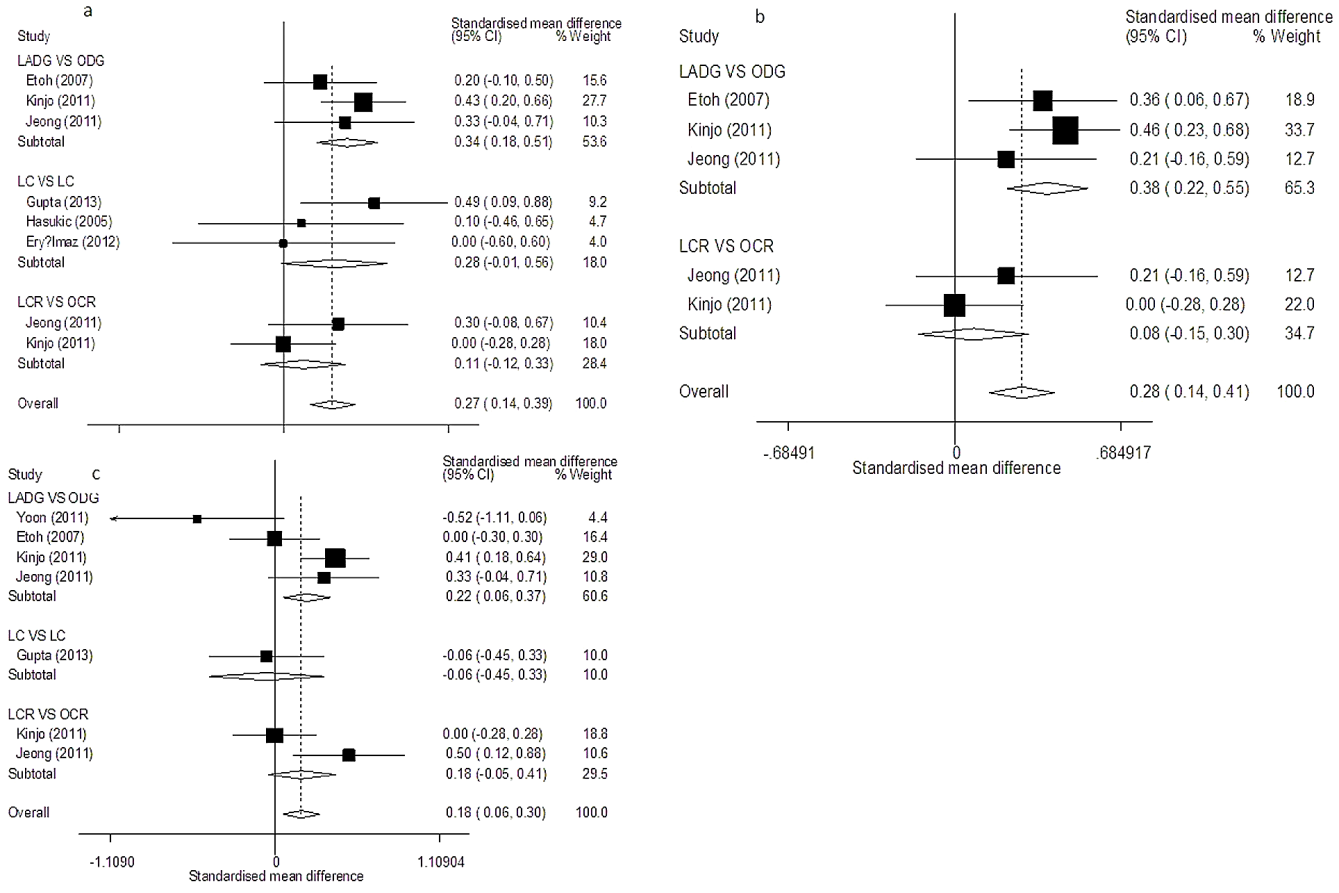
Forest plots of postoperative TB results in subgroup analysis. (a: day 1; b: day 3; c: day 7).

(Fig. 5, Table 2C) On postoperative days 1, 3, and 7, only the LADG versus ODG subgroup analysis showed a significant TB elevation. However, the overall pooled estimates continued to show significant differences between study and control groups. In these analyses, lower heterogeneity was seen across all trials, except for the results on postoperative day 7. No publication bias was evident in the studies (*P*>0.05).

### Sensitivity analysis

The inclusion criteria for this meta-analysis were subjected to a sensitivity analysis to determine whether modification of the inclusion criteria of the meta-analysis affected the results (Fig. 3a). A single study involved in the meta-analysis was deleted each time to assess the influence of each individual data set on the pooled SMDs. The corresponding pooled SMDs were essentially unaltered (data not shown), thereby indicating that our results were statistically sound.

### Risk of publication bias

Funnel plots of the studies included in our outcome analysis of the postoperative results regarding ALT, AST, and TB were prepared to assess publication bias. The funnel plot showed the CI and effect estimate. The latter showed a symmetrical distribution around the effect estimate, thereby indicating that publication bias was likely minimal for the results evaluating postoperative liver function in patients who underwent laparoscopic abdominal surgery (*P*>0.05).

However, publication bias was evident in the ALT (*P* = 0.02, Fig. 3b) and AST results (*P* = 0.04, Fig. 3c) on postoperative day 3. This may have been because of the limited number of studies that evaluated these outcomes on postoperative day 3. The funnel plot should be interpreted with some caution. Linear regression analysis was not conducted to determine funnel plot asymmetry, because none of the dichotomous outcomes were included in a sufficient number of trials for this method.

## Discussion

Presently, the association between the duration of CDP in laparoscopic abdominal surgery and hepatic injury is not fully understood. Increasing evidence suggests that the duration of CDP may be associated with reduced hepatic blood flow and induce hepatic injury. However, other studies have suggested that elevated liver enzyme values may be caused by direct liver manipulation or aberrant hepatic artery ligation rather than CDP [16]. Brundell *et al.* even suggested that duration of CDP had *no* effect on hepatic blood flow [28]. Our meta-analysis of 11 comparative studies provides evidence that the duration of CDP in laparoscopic abdominal surgery is associated with hepatic injury.

“The higher the pressure, the better the view” used to be the axiom of surgeons who needed adequate exposure for laparoscopic procedures. To provide good exposure of the surgical field, generally 10 to 15 mmHg pressure ranges are used during pneumoperitoneum [27], [29], [30]. However, when CDP is created, there may be some side effects [31], [32]. The primary mechanism for elevation of liver enzymes after laparoscopic abdominal surgery is likely due to the increased intra-abdominal pressure and its effects on portal venous flow. The 11–15 mmHg of CO_2_ that is used is higher than the normal portal blood pressure of 7–10 mmHg, which results in reduced portal blood flow [33], [34], [35], [36]. On this occasion, free radicals are generated at the end of a laparoscopic procedure, possibly as a result of an ischaemia-reperfusion phenomenon induced by the inflation and deflation of the pneumoperitoneum [27]. Free radicals can damage tissues and organs, particularly the Kupffer and the endothelial cells of the hepatic sinusoids [37]. Therefore, the elevated intra-abdominal pressure due to pneumoperitoneum may be responsible for the increase in liver enzymes after laparoscopic abdominal surgery. However, other unmeasured confounding factors may also be responsible for elevation of liver enzymes, such as extended liver traction in large laparoscopy procedure that lead to hepatocyte damage [16], and the intraoperative “squeeze” pressure effect on the liver that free liver enzymes into the blood stream [6]. This mechanism remains to be further confirmed in animal models.

Generally, this effect might be positively correlated with the pressure of CDP and its duration [20]. For example, the higher elevation of hepatic transaminases after LGBP (six-fold) than LC (two-fold) may reflect the longer operative time for LGBP [14]. However, not all results demonstrated this trend. This heterogeneity is a problem in the interpretation of the results as the studies were very heterogeneous (Table 2).

We performed a sensitivity analysis on the subgroup analyses. The LCR versus OCR subgroup analysis always produced contrary results. The study conducted by Kinjo *et al.* examined a lower pressure of CDP (8 mmHg) in the subgroup analysis and included a larger population (*n* = 324) in the study group than other studies (Table 1) [15]. However, omitting this study did not yield discordant postoperative results, eliminate the statistical significance of the results (*P*>0.05), or reduce the degree of heterogeneity (*I^2^* = 70.7%). Sensitivity analysis showed that the studies conducted by Guven *et al.*, Etoh *et al.*, and Sakorafas *et al.* exerted a major impact on our results [8], [11], [27]. Omitting these three studies from the analysis did not increase the trend seen with regard to the relationship between the duration of CDP and postoperative liver function nor did it eliminate the statistical significance of the results. The control groups in three studies included those who underwent laparoscopic surgery with low-pressure CDP (7–10 mmHg) [7], [9], [10], whereas the remaining studies included those who underwent open surgery. The results for the cohorts were analyzed separately in the subgroup analysis. However, no trend toward higher statistical significance in the laparoscopic surgery group versus the open surgery group was found.

Explanations for these differing results of the subgroup analyses may include that the small numbers of cases in some studies and the limited number of trials in the subgroup analyses increased the possibility that chance alone accounted for the results. Additionally, the CDP used for patients in the study groups was not uniform. For example, three studies involved LCR versus OCR, and these showed inconsistent results [6], [15], [16]. However, the study conducted by Kinjo *et al.* involved 8 mmHg CDP for patients in the study group, which is approximately equal to the normal portal blood pressure (7–10 mmHg) [15]; thus, hepatic blood flow may not have always been reduced intraoperatively. In contrast, in the two other studies, the CDP pressure was 12–14 mmHg in the study groups [6], [16], thereby leading to reduced hepatic blood flow. Our meta-analysis showed that the postoperative liver enzyme elevation was significant in “small” laparoscopic surgical procedures, but that a significant difference was rarely found in “larger” surgeries, such as LGBP versus OGBP. Thus, it is possible that the potential mechanisms for the postoperative elevation of liver enzymes after laparoscopic surgery in these procedures are not limited to reduced portal blood flow due to CDP; they may also include the effects of anesthetic drugs and/or local hepatic parenchymal injury from the mechanical retraction of the left lobe of the liver [14], especially given that the open control groups in these studies showed similar side effects. This was demonstrated by Clarke *et al.* [38], who found that liver enzymes increased two-fold after open gastric and biliary surgery, but that no change was seen after “small” surgical procedures, such as cystoscopy and a superficial biopsy.

We included several abdominal surgeries and then performed subgroup analyses regarding the duration of CDP according to type of surgery or control group. There is sufficient evidence in the 11 comparative studies to conclude that the duration of CDP is associated with the induction of hepatic injury in patients undergoing laparoscopic abdominal surgery.

To the best of our knowledge, this is the first reported meta-analysis to systematically evaluate the relationship between the duration of CDP and hepatic injury and to demonstrate a significant effect in this regard. Despite the variation in the results of individual studies, the accumulating evidence and large sample provided the statistical power to produce more precise and reliable efficiency estimates. Overall, the published evidence supports the assumption that the duration of CDP is associated with the induction of hepatic injury. Therefore, surgeons should pay attention to the following take home messages. First, surgeons should be cautious before planning to perform laparoscopic abdominal surgery in patients with known hepatic insufficiency. Second, we recommend using low pressure CDP (10 mmHg or lower), particularly for those undergoing prolonged laparoscopic surgery. This is because low-pressure CDP is not only feasible for decreasing hemodynamic variations but also add on to already existing benefits of endoscopic surgery [7]. Third, the potential occurrence of hepatic dysfunction should be carefully monitored after laparoscopic abdominal surgery in patients with chronic hepatic disease.

Our meta-analysis has several limitations. First, the interesting aspect of our study is the significance of this transient abnormality of liver enzymes and its clinical impact. Most studies reported that a transient increase in serum transaminase activities was most prominent on postoperative day 1, but returned to normal levels within several days after surgery [6], [8], [16], [29], [39]. Further, in some studies [8], elevated liver enzymes are not always consistently elevated; significant elevations after high-pressure CDP laparoscopic surgery compared with low-pressure CDP laparoscopic surgery or open surgery has been defined for only AST and ALT levels. Additionally, these liver enzyme alterations observed after laparoscopic abdominal surgery have not been reported to be clinically important in some studies [6], [20], [27], [29]. Since most of the studies included in our meta-analysis [6], [7], [8], [10], [11], [14] mentioned preoperative normal serum liver enzyme values as the most common reason for inclusion for patients, we cannot precisely conclude that these enzyme changes reflect a true hepatic injury in these patients, but this may be more relevant in patients with chronic liver disease like cirrhosis. This aspect needs to be investigated in future. Although the clinical importance of these enzyme elevations has not been fully clarified, surgeons should be cautious, as we suggested earlier. Second, the study design, type of CDP used for patients in the study groups, and inclusion and exclusion criteria were frequently not uniform (Table 2), thereby leading to the potential for bias. Third, several included studies had small sample sizes [7], [9], [10], [13], [14], and the types of surgery were limited. Thus, larger-scale, multi-center trials focusing on other types of surgery, such as laparoscopic appendectomies and hysterectomies, are required to provide evidence that the duration of CDP can be associated with the induction of hepatic injury. Fourth, substantial heterogeneity was present among the studies, and residual confounding is a concern. Uncontrolled or unmeasured confounding factors, such as the effects of anesthetic drugs and local hepatic parenchymal injury from mechanical retraction of the left lobe of the liver, can potentially produce biases. Unfortunately, several studies did not fully describe all their procedures. Furthermore, because currently available data on the effects of the duration of CDP in laparoscopic surgery are sparse, we were unable to assess this fully.

## Conclusions

The current evidence suggests that the duration of CDP during laparoscopic abdominal surgery may be associated with hepatic injury. Additional large-scale, randomized, controlled trials are urgently needed to further confirm this.

## Supporting Information

Checklist S1 PRISMA 2009 Checklist
(DOC)
